# Using Diagnostic Ultrasound to Support the Diagnose Sarcopenia in Older Adults: A Systematic Review

**DOI:** 10.1093/geroni/igab046.3548

**Published:** 2021-12-17

**Authors:** Edgar Vieira, Lily Charles, Monica Cortes, Tabitha Lees

**Affiliations:** Florida International University, Miami, Florida, United States

## Abstract

Dual-energy x-ray absorptiometry (DXA) is currently the gold standard for diagnosing loss of muscle mass in older adults (a component of sarcopenia diagnosis). Magnetic resonance imaging (MRI) and computed tomography (CT) have also been used successfully. Due to elevated costs, limited access, exposure to radiation, and increased difficulty of operation, other methods have been explored as alternatives. We reviewed the literature on the use of diagnostic ultrasound to assist in the diagnose sarcopenia in older adults by searching MEDLINE, Embase, and CINAHL using a variation of terms related to “ultrasound”, “sarcopenia”, and “older adults”. We included studies that included older adults over the age of 60. Eighteen studies were included after screening for eligibility and conducting full-text reviews. The most common transducer head frequency utilized in the studies were 5-12 and 8 MHz (three studies each), followed by 5, 6, and 7.5 MHz (two studies each). The most common musculature examined was anterior thigh musculature, followed by muscles of the lower leg, upper extremity, abdominals, and head/neck. Measurements most taken were muscle thickness/cross sectional area (18 studies), followed by muscle echogenicity (9 studies), and pennation angle (3 studies). Ultrasound is a reliable and valid tool to examine muscle thickness to assist in diagnosing sarcopenia. However, echogenicity measures of a muscle were not reliable. Further research is needed with increased sample size and variance amongst subjects to generalize and create normative data. In addition, standardized protocols for the use of ultrasound to assist in the diagnosing sarcopenia need to be established.

